# Novel early life risk factors for adult pulmonary hypertension

**DOI:** 10.1177/2045894019842002

**Published:** 2019-04-16

**Authors:** Kara N. Goss, Eric D. Austin, Therese J. Battiola, Robert S. Tepper, Tim Lahm

**Affiliations:** 1 Division of Allergy, Pulmonary and Critical Care, Department of Medicine, University of Wisconsin School of Medicine and Public Health, Madison, WI, USA; 2 Division of Allergy, Immunology and Pulmonary Medicine, Department of Pediatrics, Vanderbilt University Medical Center, Nashville, TN, USA; 3 Section of Pediatric Pulmonology, Allergy and Sleep Medicine, Department of Pediatrics, Indiana University School of Medicine, Indianapolis, IN, USA; 4 Division of Pulmonary, Critical Care, Sleep and Occupational Medicine, Department of Medicine, Indiana University School of Medicine, Indianapolis, IN, USA; 5 Richard L. Roudebush VA Medical Center, Indianapolis, IN, USA

**Keywords:** prematurity, oxygen, infection, developmental origins

## Abstract

The role of perinatal insults in the development of adult onset pulmonary hypertension (PH) is unclear. We surveyed patients with and without PH for a history of early life risk factors, and identified prematurity, oxygen use, and respiratory illness each as risk predictors for development of adult PH.

## Introduction

Adult-onset pulmonary hypertension (PH) is thought to result from the coalescence of multiple risk factors such as genetic predisposition or hormonal influences.^1^ However, the role of early life risk factors in priming the pulmonary vasculature for later dysfunction remains poorly defined.^2^ We hypothesized that adult PH patients would demonstrate a higher prevalence of early life risk factors such as premature birth, treatment with oxygen therapy in infancy, or significant lower respiratory infection in early childhood, and surveyed adults with and without a history of PH for these early life risk factors.

## Methods

Adults aged 18–60 years with a diagnosis of PH, including all five groups, were recruited from Indiana University Health (IUH) PH clinics or during attendance at the 2014 Pulmonary Hypertension Association (PHA) Conference (Indianapolis, IN, USA) and asked to complete a brief survey. Survey questions included the following:

1. Were you born *4 weeks or more* premature? If yes, how premature?
2. Did you receive any form of oxygen in your *first 6 months* after birth? If yes, please describe.
3. Did you have any hospitalizations for a respiratory illness in your *first five years* of life? If yes, please describe.

Each question offered response choices of “yes,” “no,” or “unsure.” Individuals responding “unsure” were excluded from further analysis for that question. For the IUH cohort, participants were identified based on ICD-9 codes for PH and paper surveys were mailed to the home address. For the PHA cohort, individuals participating in the Research Room at the PHA Conference were asked to complete the survey. A diagnosis of PH was confirmed by chart review for all patients and baseline PH characteristics were obtained from medical records. To establish expected rates of early life risk factors, adults aged 18–60 years with no self-reported history of PH were recruited from a general medicine clinic at the University of Wisconsin and asked to complete the same survey. Surveys were approved by the IU and UW Institutional Review Boards; all individuals provided informed consent. For binary survey responses, odds ratios (OR) were initially calculated by Chi-square analysis, with confidence intervals computed with the Woolf logit method (Prism GraphPad, La Jolla, CA, USA). To ensure that the early life effects were not significantly influenced by age or race, a logistic regression to predict PH after adjusting for age and race (white or non-white) was performed using R (Foundation for Statistical Computing, Vienna, Austria). Hemodynamic variables were compared using unpaired t-tests. Tests were two-tailed; a *P* value < 0.05 was considered significant.

## Results

Among IUH patients mailed a paper survey, 43 responded (26% response rate). Another 56 patients participated at the 2014 PHA Research Room (response rate of 22%). The majority of patients had Group 1 PH. An additional 100 individuals without PH served as controls. PH patients were non-significantly older and more likely to be minorities (PH: 46.6 years vs. non-PH: 43.8 years, *P* = 0.06; PH: white 78% vs. non-PH: 87% white, *P* = 0.12).

Regarding prematurity, 10.11% of PH individuals reported a history of preterm birth (Table 1), with an average gestational age of 33.9 ± 0.6 weeks (range = 32–36 weeks). For comparison, only 2.2% of controls reported a history of preterm birth (OR = 5.06, 95% confidence interval [CI] = 1.06–24.14). When adjusted for age and race, prematurity remained a significant predictor of adult PH (OR = 5.90, 95% CI = 1.44–39.94).

**Table 1.** Survey responses among patients with and without pulmonary hypertension (PH).

	Adults without PH		Adults with PH		OR	95% CI	*P* value
	Yes (n (%))	No (n (%))	Yes (n (%))	No (n (%))			
Born premature?	2 (2.2)	90 (97.8)	9 (10.1)	80 (89.9)	5.06	1.06–24.14	0.025
Oxygen in first 6 months?	3 (3.3)	87 (96.7)	11 (12.0)	81 (88.0)	3.94	1.06–14.63	0.029
Respiratory hospitalization in first 5 years?	4 (4.2)	92 (95.8)	11 (12.8)	75 (87.2)	3.37	1.03–11.03	0.035

Similarly, PH patients were more likely to report a history of early oxygen therapy (12.0% vs. 3.3%; OR = 3.94, 95% CI = 1.06–14.63), which remained significant when adjusted for age and race (OR = 5.13, 95% CI = 1.39–24.89). Indications included premature birth (n = 3), acute respiratory illness (n = 4), congenital heart disease (n = 3), and unreported (n = 1).

Adult PH patients were also more likely to report early hospitalizations for respiratory illness (12.8% vs. 4.2%; OR = 3.37, 95% CI = 1.03–11.03), which also remained significant when adjusted for age and race (OR = 4.69, 95% CI = 1.34–21.94). Indications for respiratory hospitalization included: pneumonia (n = 5); severe asthma; croup; “respiratory infection for two weeks at five weeks of age;” “cyanosis;” “bronchitis as infant;” and “unknown” (n = 1 each). There was not a significant overlap between history of prematurity or respiratory hospitalization, as only two individuals responded “yes” to both. However, early oxygen use was commonly seen in both individuals born preterm and infants requiring respiratory hospitalization.

When analysis was limited to patients with pulmonary arterial hypertension (PAH; Group 1 PH), similar rates of premature birth (9.59%) and early life hospitalizations (11.59%) were detected, though early treatment with oxygen was non-significantly higher among PAH patients (17.6%). Comparison of PH characteristics did not identify any differences among those with and without specific early life events (Table 2).

**Table 2.** Comparison of PH characteristics among patients with and without a history of early life events.

	History of preterm birth?		History of oxygen treatment in first 6 months?		History of hospitalization for respiratory illness?	
	Term	Preterm	No Oxygen	Oxygen	Non-hospitalized	Hospitalized
*Demographics*	n = 80	n = 9	n = 81	n = 11	n = 76	n = 10
Age (years)	46.7 ± 1.1	43.8 ± 4.6	47.2 ± 1.1	41.7 ± 3.3	47.2 ± 1.1	43.9 ± 3.0
Female gender (n (%))	64 (80)	8 (88.9)	63 (77.8)	11 (100)	61 (80.3)	9 (90)
*WHO classification*	n = 80	n = 9	n = 81	n = 11	n = 76	n = 10
Group 1 (n (%))	66 (82.5)	7 (77.8)	65 (80.2)	9 (81.8)	61 (80.3)	8 (80)
Groups 2–5 (n (%))	14 (17.5)	2 (22.2)	16 (19.8)	2 (18.2)	15 (19.7)	2 (20)
*Hemodynamics*	n = 54	n = 8	n = 58	n = 6	n = 53	n = 9
sPAP (mmHg)	77.4 ± 2.9	69.6 ± 7.2	76.3 ± 2.9	67.7 ± 11	75.6 ± 3.1	75.1 ± 5.9
dPAP (mmHg)	32.8 ± 1.2	29.8 ± 4.6	31.8 ± 1.2	25.8 ± 3.7	31.6 ± 1.3	32.2 ± 2.6
mPAP (mmHg)	50.3 ± 1.5	44.4 ± 4.5	49.5 ± 1.5	39.7 ± 6.9	48.73 ± 1.7	47.7 ± 3.3
PAWP (mmHg)	10.9 ± 0.9	12.4 ± 1.4	11.4 ± 0.9	11.2 ± 1.3	11.2 ± 0.8	9.1 ± 2.3
PVR (WU)	9.6 ± 0.6	7.8 ± 1.1	9.2 ± 0.6	6.5 ± 1.3	9.0 ± 0.6	9.0 ± 1.5
CO (L/min)	4.9 ± 0.2	5.1 ± 0.7	5.0 ± 0.2	5.2 ± 0.7	5.1 ± 0.3	4.6 ± 0.6
*Functional class*	n = 79	n = 9	n = 80	n = 11	n = 75	n = 10
NYHA 1–2 (n (%))	29 (36.7)	5 (55.6)	32 (40)	7 (63.6)	30 (40)	5 (50)
NYHA 3–4 (n (%))	50 (63.3)	4 (44.4)	48 (60)	4 (36.4)	45 (60)	5 (50)

*P* > 0.05 for all comparisons within each survey question.

sPAP, systolic pulmonary arterial pressure; dPAP, diastolic pulmonary arterial pressure; mPAP, mean pulmonary arterial pressure; PAWP, pulmonary artery wedge pressure; PVR, pulmonary vascular resistance; CO, cardiac output; NYHA, New York Heart Association.

## Discussion

Here, we have identified premature birth, early oxygen treatment, and early hospitalization for respiratory illness as risk factors for the development of adult PH. Of these, premature birth appears to be the strongest risk factor. Among adults born extremely premature with an average gestational age of 29 weeks, one recent study demonstrated that 45% have borderline or overt resting PH, with a group average mean pulmonary arterial pressure (mPAP) of 20 mmHg.^3^ Further, these individuals have an exaggerated rise in PAP with exercise, further supporting the presence of subclinical pulmonary vascular dysfunction.^3,4^ Although the severity of prematurity observed in our study was only mild to moderate, our observed 5.06-fold increased risk for PH among adults born premature is similar to the 3.08-fold higher risk for PAH development among adults born premature identified in a recent small Swedish adult PAH Registry study.^5^ Among adolescents born premature, the risk for PAH was 8.46-fold higher.^6^ By adjusting for known factors associated with PH, the Swedish authors suggested that there are additional unknown neonatal factors beyond prematurity that increase the risk for adult PH.^5,6^

Intriguingly, we newly identify early life oxygen therapy and hospitalization for respiratory illness as potential novel risk factors for adult onset PH. Although longitudinal studies have suggested that early childhood pneumonia is associated with an increased risk for adult chronic obstructive pulmonary disease, to our knowledge, an increased incidence of early respiratory illness has not previously been reported among PH patients.^7,8^ Given that the developing lung requires growth and maturation of both the alveolar and vascular spaces in tandem, it seems plausible that factors impairing long-term alveolar health may also affect the vasculature; animal studies support this concept.^9^ The role of early oxygen exposure in altering long-term pulmonary vascular function is unclear. Whether early oxygen exposure causes intrinsic pulmonary vascular dysfunction or merely is a confounder given its use in treating both prematurity and respiratory infection is unknown. However, in rodents, neonatal hyperoxia exposure alone, without true premature birth or respiratory infection, results in the development of PH and may not present with clinically relevant pulmonary vascular disease until adulthood.^10^

Study limitations include the retrospective nature and potential for recall bias, though this would apply to both PH and non-PH participants. However, the indications provided for oxygen use and respiratory hospitalizations seemed appropriate and less likely to represent simple emergency room visits. Furthermore, the degree of prematurity observed in this study was relatively mild. The extent to which extreme preterm birth has a greater effect on the lifetime risk for developing PH remains to be determined, as the first wave of survivors of extreme prematurity are just now in early adulthood. Finally, although we have a relatively small sample size in this survey, we note that our survey sample size for PH patients is still 1.6 times larger than reported in the Swedish registry study, with overall similar ORs.^5^

In conclusion, we have identified premature birth, neonatal oxygen treatment, and childhood hospitalization for respiratory illness as risk factors for the development of adult PH. These initial and hypothesis-generating results could serve as the rationale and basis for developing prospective and mechanistic investigations in the future. Specifically, large registry studies with access to birth and early life hospitalization records are needed to fully assess early life risk factors for development of PH. Evaluation should include additional risk factors such as presence of pre-eclampsia, extreme prematurity, left to right shunts such as a patent ductus arteriosus, and both bacterial and viral respiratory infections. Finally, prospective childhood and adult PH registries should also seek to capture data regarding early life risk factors to identify if these events have implications for disease prognosis or response to therapy.
